# The role of hormones in attraction and visual attention to facial masculinity

**DOI:** 10.3389/fpsyg.2023.1067487

**Published:** 2023-02-13

**Authors:** Ray Garza, Jennifer Byrd-Craven

**Affiliations:** ^1^Department of Psychology and Communication, Texas A&M International University, Laredo, TX, United States; ^2^Oklahoma Center for Evolutionary Analysis, Department of Psychology, Oklahoma State University, Stillwater, OK, United States

**Keywords:** eye-tracking, mate choice, sexual selection, ovulatory shift hypothesis, attention, attraction

## Abstract

The current study investigated the ovulatory shift hypothesis, which suggests that women prefer more masculine traits when estradiol is high, and progesterone is low (E/P ratio). The current study used an eye tracking paradigm to measure women’s visual attention to facial masculinity across the menstrual cycle. Estradiol (E) and progesterone (P) were collected to determine if salivary biomarkers were associated with visual attention to masculine faces in a short- and long-term mating context. Women (*N* = 81) provided saliva samples at three time points throughout their menstrual cycle and were asked to rate and view men’s faces that had been manipulated to appear feminine and masculine. Overall, masculine faces were viewed longer compared to feminine faces and this was moderated by mating context, where women viewed masculine faces longer for a long-term relationship. There was not any evidence suggesting that E/P ratio was associated with preferences for facial masculinity, but there was evidence to suggest that hormones were associated with visual attention to men in general. In line with sexual strategies theory, there was evidence to suggest that mating context and facial masculinity are important in mate choice; however, there was no evidence to suggest that women’s mate choice was associated with shifts across the menstrual cycle.

## 1. Introduction

According to sexual selection theory, members of one sex should be sensitive to cues that advertise important reproductive information about the opposite sex in mate choice (Andersson, 1994). In sexually reproducing species, male ornamentation has evolved due to a variety of factors, such as ecological influences, within-sex competition, and through female choice. In addition, female preferences for exaggerated secondary sexual characteristics have evolved because of indicator mechanisms that signal high heritability and direct phenotypic benefits, such as protection and parental ability (Andersson, 1994). In human males, exaggerated secondary sexual characteristics, such as facial masculinity, are preferred by some women (DeBruine et al., 2006; Boothroyd et al., 2013; Marcinkowska et al., 2018b) perhaps due to its association with immunocompetence (Zahavi, 1975; Folstad and Karter, 1992; Thornhill and Gangestad, 1999a, 1999b; Little et al., 2011a; Rantala et al., 2012). Some suggest that women should have a stronger preference for sexually dimorphic traits in men when conception is optimal (Gangestad and Thornhill, 1998; Gangestad and Haselton, 2015). However, ovulation in human populations is concealed to potential mates and to the female herself (Alexander and Noonan, 1979; Alexander, 1990), which may indicate that preferences across the menstrual cycle may not be under direct conscious awareness. Methods that are sensitive in detecting subtle behavioral changes, such as eye movements, can demonstrate that women display implicit attentional biases toward masculinity. This study uses an eye tracking paradigm to investigate whether preferences for facial masculinity are influenced by reproductive hormones throughout the menstrual cycle.

Both men and women have faced adaptive challenges in mate selection (Buss and Schmitt, 1993, 2019). Humans have evolved psychological adaptations in response to many facets of mate selection. For women, identifying which men will make good partners has been one of those challenges, as women have had to discern which mates will provide them with indirect benefits in the form of high-quality genes for their offspring, direct benefits in the form of resource protection and status transmission, and/or a mate who is invested in long-term pair bonding. Since women have more to lose in making a poor mate choice, and given the long, intensive parenting effort of human life histories (Flinn et al., 2007), assessing these features in human mating are important. Women have been known to place premiums on characteristics that signal good genes, such as attractiveness and health (Buss, 1989; Cashdan, 1996) and characteristics that signal immediate resource transmission and future resource acquisition (Cashdan, 1996). One principle of sexual strategies theory is that women pursuing men with these features would have obtained benefits from short-term mating, as pursuing men with these features for long-term relationships would put women and their children at risk for abandonment and increased competition from other women (Buss and Schmitt, 1993, 2019). Since short-term mating requires minimal investment, benefits obtained (i.e., superior genes, resources) would have had to outweigh the costs of not pursuing a mate for a long-term commitment.

Facial cues are directly observable characteristics that provide women hormonal information about men. Androgen levels are associated with exaggerations of secondary sexual characteristics, such as the brow ridges and jaw (Thornhill and Gangestad, 1996). Advertisement of these traits should signal indirect benefits (i.e., good genes) to women because of testosterone’s immunosuppression. Using this framework, research has demonstrated that women tend to prefer masculine over feminine faces (Johnston et al., 2001; DeBruine et al., 2006; Cornwell and Perrett, 2008; Little et al., 2008; Boothroyd et al., 2013) and find them sexually attractive (Marcinkowska et al., 2018b). Masculine faces have been associated with immunocompetence (Rhodes et al., 2003; Rantala et al., 2012), disease resistance (Thornhill and Gangestad, 2006), and strength (Windhager et al., 2011). Facial masculinity has also been associated with direct benefits, such as protection and resource acquisition (Sell et al., 2009). Men with masculine characteristics display more muscularity, dominance, and physical strength (Fink et al., 2007; Puts, 2010; Windhager et al., 2011). However, there is a tradeoff between securing a mate with good genes or securing a mate that is willing to invest in offspring (Marcinkowska et al., 2018a). Men with masculine features are perceived as less parentally investing (Perrett et al., 1998), and more willing to engage in short-term relationships (Rhodes, 2006; Boothroyd et al., 2008). Therefore, preferences for masculinity may be calibrated to maximize the likelihood and need of obtaining indirect (i.e., good genes) or direct benefits (i.e., resource acquisition, protection).

The ovulatory shift hypothesis (Gangestad and Thornhill, 1998) suggests that shifts in women’s hormones across the menstrual cycle are associated with shifting sexual preferences. It asserts that this heightened preference would have been associated with increased reproductive success in ancestral females compared to those who did not exhibit a cyclic shift in preferences (Gildersleeve et al., 2014). It would also indicate that women’s preferences would be attenuated outside of the fertile window due to the cost of losing social mates or in choosing the wrong mate (Gangestad et al., 2005). Research into fertility status in women’s mate preferences has suggested women prefer men who are symmetrical because of its association with health and immunocompetence (Thornhill and Gangestad, 1999a). Women in the high fertility phase display preferences for men with masculine faces compared to women who are at the low fertile phase of the menstrual cycle (Penton-Voak and Perrett, 2000; Little et al., 2007; Dixson et al., 2018a). However, other studies have not shown an ovulatory shift for preferences to men’s masculine characteristics. Cyclic shifts did not play a role in rating men’s facial and body masculinity (Marcinkowska et al., 2018c). Women during the fertile phase of the menstrual cycle have shown to rate all men as equally attractive, regardless of masculine traits (i.e., waist to chest/shoulder ratios) (Garza et al., 2017; Jünger et al., 2018a; Garza and Byrd-Craven, 2019). Other research on male traits signaling masculinity (i.e., degree of hair distribution and beardedness) (Gangestad and Thornhill, 2008) have not shown that women shift their preferences for these traits across the fertile period (Rantala et al., 2010; Dixson and Rantala, 2016; Garza et al., 2017; Dixson et al., 2018b). In examining vocal masculinity, women’s preferences for masculine voices were not associated with ovulatory status (Jünger et al., 2018b).

One issue in research investigating ovulatory shifts in men’s mating preferences has been the accuracy of methods used. Traditionally, self-report methods using the calendar method, where women count the days from their previous menstrual period, have been used to dichotomize women into low or high fertility conceptive probability. The use of precise methods in detecting fertility status have become more common practice. During the pre-ovulatory phase of the menstrual cycle, women experience a rise in estradiol and a decline in progesterone (Roney and Simmons, 2013). By tracking the ratio of estradiol to progesterone, it is possible to determine the increased likelihood of ovulation, and if women’s shifts for specific mate preferences were to occur, they should occur during a rise in the E/P ratio. The estradiol to progesterone ratio (i.e., E/P Ratio) is a recommended method in detecting fertility status (Blake et al., 2016; Gangestad et al., 2016) because during the late-follicular phase estradiol is expected to be higher than progesterone than any other point in the menstrual cycle, therefore higher values are an indicator of increased likelihood of ovulation, while lower values are an indicator of post-ovulation. Findings for hormonal shifts in preferences for masculinity have been equivocal. Preferences for masculinity have been associated with increased levels of estradiol (Roney and Simmons, 2008), while others have not found any preferences using luteinizing hormone (Peters et al., 2009). Recent studies on mate preferences for masculine faces using the E/P ratio have not revealed a preference during peak fertility (Marcinkowska et al., 2016, 2018a). Progesterone has shown to be predictive of masculinity preferences as a function of relationship status (Marcinkowska et al., 2018a). Partnered women’s progesterone levels were related to a weaker preference for masculinity. Since increased progesterone levels are associated with pregnancy (Gilbert, 2000), when progesterone is high, women may direct their attention to parenting instead of honest signals of good genes (Marcinkowska et al., 2018a). Although these studies have relied extensively on preferences tasks, such as choosing which face (i.e., masculine vs. feminine) is preferred, it is not yet understood if there are any hormonal influences in the way that women process these features visually.

Although previous research has depended on women’s stated preferences for masculinity, eye tracking has provided researchers with a behavioral measurement of these preferences. Eye tracking is a sensitive gaze contingency technique that provides real-time visual processing, such as implicit and explicit measurements. Eye tracking procedures are advantageous over traditional preference tasks, as they are less susceptible to experimenter expectancy, they correlate with self-reported preferences, and they provide information to smaller features (i.e., regions of interest) that can help investigate the nuances associated with mate preferences (Krupp, 2008). Eye tracking research in women’s assessments of sexually dimorphic (i.e., masculine/feminine) faces has been limited. In using both male and female participants in a Chinese sample, Wen and Zuo (2012) showed that participants fixated first at masculine faces, suggesting that masculine features are important in early visual processing. However, since their research combined data from both men and women in tracking their visual movements, it is likely that there were differences in the interpretation of masculine vs. feminine faces (i.e., men may perceive men as dominant/aggressive). In a similar study, albeit using masculinity and attractiveness, Yang et al. (2015) found that masculine faces were preferred for first fixation duration and total visual time, but it was dependent on their level of attractiveness. In using female participants only, Burris et al. (2014) found that women focused their visual attention to feminine rather than masculine faces using visual metrics that account for early stage (i.e., first fixation duration) and late stage (i.e., total dwell time) processing. However, given the differences that exist between Chinese and European populations in masculinity preferences, it can be argued that there are cultural differences in preferences, as both studies using Chinese populations found preferences for masculinity in eye tracking designs, while in a European sample, preferences were for feminine faces. Although not explored, one possible explanation for preferences in the Chinese and European study could be the role of population density. Denser populations may rely on heuristics and exaggerated dimorphic characteristics due to the frequent exchange of social encounters (Scott et al., 2014; Marcinkowska et al., 2018b).

The current study aimed to address issues raised by previous studies investigating the ovulatory shift hypothesis by using an eye tracking paradigm to track women’s visual preferences to sexually dimorphic faces. Although recent studies have not found an effect for fertility status using a forced choice task in preferences, the current study addressed whether shifts in hormones affect women’s visual preferences by tracking eye movements across different times of the menstrual cycle. This study addressed the following research questions: (1) Do hormones moderate the relationship between sexual dimorphism and attraction/visual attention? It is hypothesized that as estradiol increases and progesterone decreases (i.e., high E/P ratio), women will rate masculine faces more attractive and view them longer, (2) Do hormones affect preferences for masculine features depending on mating context? As a direct test of the sexual strategies theory (Buss and Schmitt, 1993), we investigate if women have evolved psychological mechanisms for short- and long-term mate preferences by tracking visual attention to masculine and feminine faces in these different contexts. Research has suggested that women prefer masculine men for short-term relationships (Little et al., 2002, 2007; Little et al., 2011b), however, tracking reproductive hormones has not been investigated using an eye tracking paradigm. It is hypothesized that women with high E/P ratios will view masculine faces longer when considering men for a short-term relationship.

## 2. Materials and methods

### 2.1. Participants

A G*Power analysis in detecting a small to moderate effect size indicated a sample size of approximately 71 participants. Further, we relied on recommendations from Gangestad et al. (2016) in estimating an appropriate sample size for a within-subjects designs testing the ovulatory shift hypothesis. Participants were 81 heterosexual women from Oklahoma State University (M_age_ = 19.27, SD_age_ = 2.83) who signed up on the university’s online participant recruitment system to participate in the three-part study in exchange for course credit. Participants were only allowed to sign up for the study if they were not on any hormonal-based birth control, were not pregnant, did not smoke, and identified primarily as heterosexual. The sample demographics were White (*N* = 55), African-American (*N* = 9), Hispanic (*N* = 5), Native-American (*N* = 5), Asian-American (*N* = 4), and Other (*N* = 2).

### 2.2. Measures

#### 2.2.1. Sexually dimorphic faces

The sexually dimorphic stimuli used were from the London Face Lab, which include a composite of masculinized and feminized faces of the same individual that have been morphed to indicate −50% femininity and + 50% masculinity (DeBruine and Jones, 2017).

#### 2.2.2. Mating context prompts

Two mating context prompts adopted from Jones et al. (2018) were used to connote information on a short-term and long-term relationship. In the short-term-attractiveness test, women were given the following information: “You are looking for the type of person who would be attractive in a short-term relationship. This implies that the relationship may not last a long time. Examples of this type of relationship would include a single date accepted on the spur of the moment, an affair within a long-term relationship, and possibility of a one-night stand.” In the long-term-attractiveness test, women were given the following information: “You are looking for the type of person who would be attractive in a long-term relationship. Examples of this type of relationship would include someone you may want to move in with, someone you may consider leaving a current partner to be with, and someone you may, at some point, wish to marry (or enter into a relationship on similar grounds as marriage).”

#### 2.2.3. Hormonal assays

On the day of salivary analysis, saliva samples were thawed for 1 ½ hrs and then they were centrifuged at 1,500 rpm’s for 15 min. Estradiol and Progesterone were assayed using enzyme linked immunosorbent assays (ELISA) following Salimetrics protocols which required a serial dilution for standards and pipetting samples in duplicates (Estradiol: 100 μL, Progesterone: 50 μL). The standards for estradiol were 32, 16, 8, 4, 2, and 1 pg/μL, and for progesterone the standards were 2,430, 810, 270, 90, 30, and pg/μL. After pipetting each plate, they were set aside for incubation for 1 h, then were washed four times using a Bio-Tek plate washer. We added a TMB solution and incubated each assay for 30 min in the dark. This was followed by adding 50 μL of the stop solution to each well and mixed for 3 min before being read using a Bio-Tek 808 lx plate reader using the Gen5 software. The intra and inter-assay coefficient (CV) for progesterone was 6.68 and 5.64%, and 7.52 and 6.15% for estradiol.

#### 2.2.4. Eye tracking metrics

Eye tracking data were recorded using a Tobii X2-60, which is a non-invasive eye tracking instrument that records eye movements at 60 frames per second (60 hz). We used three eye tracking metrics, first fixation duration, total visit duration, and number of fixations. First fixation duration was defined as the duration of the first fixation on an interest area (i.e., feminine, masculine face) in milliseconds (ms), and it is often used as an eye tracking metric to indicate saliency upon first view or early-onset processing (Conklin et al., 2018). For late-onset processing, total visit duration and number of fixations were used. Total visit duration measures the duration of time spent on an interest area for each trial, and it is often used as a measure of late-onset or effortful processing (Conklin et al., 2018). The number of fixations (i.e., fixation count) is an eye tracking metric often used to complement total visit duration and it is an alternative method of considering attention (Conklin et al., 2018). The number of fixations is defined as the number of times a fixation is made on an interest area (i.e., feminine, masculine faces). Therefore, first fixation duration can be considered a measurement of early- or automatic processing, while total visit duration and number of fixations can be considered late- or effortful levels of processing. A fixation is a period in which a non-moving stimulus is being viewed (Holmqvist et al., 2011), which is then followed by a saccade. Interest areas were created using the Tobii Lab Pro interest area creator, and we created two interest areas per trial, an interest area encompassing a masculine face and an interest area encompassing a feminine face. The regions of interest included the outline of the faces (i.e., ears, forehead, jawline), and excluded the shoulders, hair, and background of the image.

### 2.3. Procedure

The institutional review board and Oklahoma State University reviewed and approved this study. Women signed up for the 3-part study using the university’s SONA online study sign-up system. The study was announced as, “Attention to Male Images,” and it included information as to the requirements to be eligible to sign up. A pre-screener was used to only sample participants who were female and primarily identified themselves as heterosexual. Participants were only allowed to sign up for the study if they had typical menstrual cycles and were not on any hormonal-based contraceptives. They were informed that before each session of the 3-part study, they could not have anything to eat 60 min prior to the study, and to choose a timeslot that was consistent throughout the sessions, such as choosing the same time for each of the three sessions. To capture variability in reproductive hormones, women were asked to participate at three different time periods within a menstrual cycle. Following from Marcinkowska et al. (2018a), participants were instructed to choose a time slot that corresponded to the early follicular phase (days 2–8), ovulation (no later than day 20), and luteal phase (last week of their menstrual cycle). As part of the demographics questionnaire, they also indicated their menstrual cycle status that corresponded to their time of visit. Participants had to wait 7-days before signing up for another session in order to prevent them from signing up for consecutive sessions and to maximize variability in their sample.

Upon entering the laboratory, participants were given a consent form which included information that was addressed on SONA, as well as additional information as to what participants could expect (i.e., demographic questions & surveys). Participants were notified that they would viewing images of men, and their task was to view the images and rate them. They were also informed of the different survey instruments that needed to be completed. Upon consent, participants provided saliva sample through passive drool collection using a saliva collection tube into a saliva collection vial (1.5 mL). All participants provided a saliva sample within 1–3 min. No participant took over 3 min in providing a saliva sample. Once saliva collection was complete, it was stored in a −80°C freezer until the day of salivary analysis.

**Figure 1:**
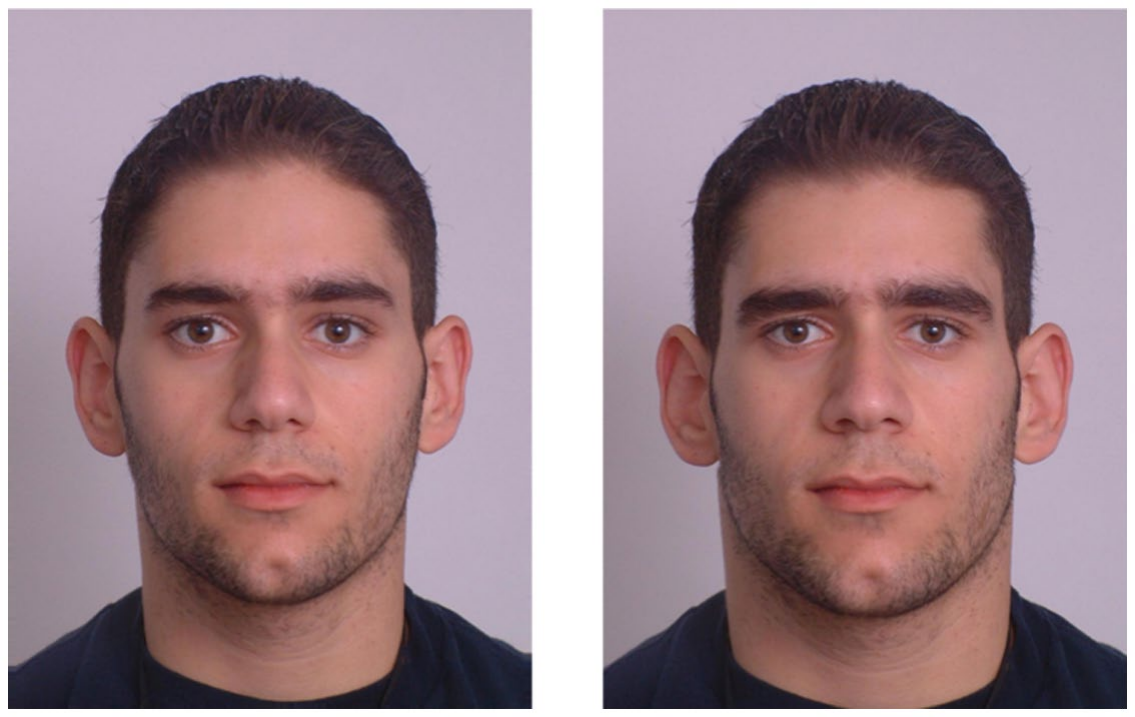
Presentation of a male face depicting a femininized (left) and masculinized (right) version. Reproduced with permission from Lisa DeBruine. Available at: https://figshare.com/articles/dataset/Young_Adult_White_Faces_with_Manipulated_Versions/4220517?file=7826521.

**Figure 2:**
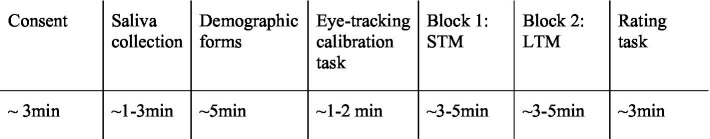
Sample timeline for each session. Blocks 1 & 2 were randomized and counterbalanced for each of the three sessions. In total, each session was approximately 25–30  min in duration.

Participants were then instructed to sit in front of a computer screen where an eye tracking device was magnetically connected to the desktop (Tobii X2-60). The eye tracker was situated within 50 cm of the participant to be able to record eye movements adequately. Before beginning the eye tracking study, participants performed a 5-point visual calibration test which consisted of following a red dot randomly across the screen to 5 positions. When complete with the calibration test, their eyes were checked again to ensure that they were centered on the computer screen, and then they were given instructions to the study. For one block, they were given instructions to view the images of men as if they were looking for a short-term relationship, defined as a relationship that consisted of a one-night stand or casual encounter where no commitment was expected. For the other block, they were instructed to view the images of men as if they were looking for a partner for a long-term relationship, defined as a committed partnership, such as marriage or a relationship lasting months. The use of the mating context prompts was adopted from Jones et al. (2018). Participants were instructed that they were to view images of men and to view the images as they would any image on a computer screen. They were presented with 20 pairs of men for two separate blocks randomly ordered to include a masculinized or feminized version of the same male on either side of the presentation (see Figure 1). They viewed each pair for 3,000 ms, followed by a fixation cross ‘X’ at the center of the screen which was presented for 500 ms before the screen refreshed and randomly presented another pair. The duration for reading each prompt and viewing 20 pairs of images was between 3 and 5 min for each block. For each of the sessions, participants did not view the blocks in the same order, and the images were counterbalanced to appear on different sides of the screen. In total, they viewed 80 images per session. A sample timeline of each session is shown in Figure 2.

Once complete with the eye tracking portion of the study, participants were instructed to rate the men for their perceived physical attractiveness using a Likert scale where ‘1 = unattractive’ to ‘7 = extremely attractive.’ They provided ratings for men under a short-term and long-term relationship context, in which they viewed each male image sequentially and not in pairs. For each image, the Likert scale rating was presented at the bottom of the screen, and when a rating was given, the screen refreshed another image. Participants were not given a time limit for the attractiveness rating. They then completed a sociodemographic questionnaire which included items about their age, ethnicity, sexual orientation, relationship status, and menstrual cycle status. For the sexual orientation question, participants indicated their sexual orientation by selecting if they identified as heterosexual, homosexual, bisexual, or prefer not to answer. For the menstrual cycle questionnaire, a forward counting method was used to indicate cycle status where participants indicated how many days had passed since the onset of menstrual bleeding. Participants were dismissed from the study and were reminded to return for the subsequent parts (i.e., time 2 & 3). They were sent follow-up emails to remind them of sessions 2 and 3. For time sessions 2, participants were instructed to return no later than the 20th day of their menstrual cycle, and for time session 3, they were instructed to return during the luteal phase of their menstrual cycle or the last week before menstruation. In total, each session was approximately 25–30 min.

## 3. Results

### 3.1. Statistical analyses

Data were analyzed using linear mixed-effects models with maximum likelihood using the packages lme4 and lmertest (Bates et al., 2015) in R for dependent variables attractiveness, first fixation duration, total visit duration, and fixation count. Linear mixed-effects models are robust multilevel models that account for variation across subjects, trials, and time varying covariates (i.e., estradiol & progesterone). Since participants were asked to rate and view multiple images across multiple time points, linear mixed effects models are recommended for analyzing designs with multiple repeated observations over time. For each session, participants viewed 80 images (i.e., 40 pairs), which totaled 240 images for all three sessions. Estradiol, progesterone, and E/P ratio were centered on their subject-specific means to interpret within effects (Brauer and Curtin, 2017). Mating context (i.e., STM, LTM), facial masculinity (i.e., feminine, masculine), estradiol, progesterone, estradiol to progesterone ratio (i.e., E/P ratio) were entered as fixed factors, and participants and trials were entered as random effects. All models met the assumptions of normality of residuals, and we performed qq-plots to test those assumptions. Outliers for visual time were screened at ±2.50 z-scores from the mean. For all of our analyses, the *R^2^* Marginal (i.e., fixed effects) and *R^2^* Conditional (i.e., random effects: subjects and trials) are reported for our total effect sizes for our models. R^2^_Marginal_ and R^2^_Conditional_ effect sizes are to be interpreted as variance accounted for. All post-hoc analyses were conducted using a Bonferroni correction.

#### 3.1.1. Descriptive statistics

Table 1 presents the descriptive statistics for women’s hormone levels, estradiol, progesterone, and E/P ratio.

**Table 1:** Raw hormone levels for estradiol, progesterone, and E/P ratio across the three sessions.

	Estradiol	Progesterone	E/P Ratio
Session 1	1.64 (1.60)	126 (85.8)	0.01 (0.007)
Session 2	1.45 (0.74)	149 (116)	0.05 (0.34)
Session 3	1.54 (1.05)	145 (93.9)	0.01 (0.008)

#### 3.1.2. Attractiveness

For attractiveness, the overall variance explained in the model was, *R*^2^_Marginal_ = 0.03, R^2^_Conditional_ = 0.34. There was a weak significant effect for progesterone, *b* = −0.0007, *SE* = 0.0002, 95%CI [−0.001, −0.0003], *p* < 0.001. When women’s progesterone was higher, ratings of attractiveness were lower. There were no other significant effects or interactions.

#### 3.1.3. First fixation duration

First fixation duration was defined as the average duration of the first fixation to a region of interest (i.e., feminine, masculine face), and it is often used as a measure indicating saliency upon first view. The overall variance explained from the model was, *R*^2^_Marginal_ = 0.004, and *R^2^*_Conditional_ = 0.17. There was a significant effect for women’s E/P ratio, *b* = −31.55, *SE* = 10.67, 95%CI [−54.06, −12.21], *p* = 0.002. When women’s E/P ratio was higher their first fixation durations were shorter. There were no other significant effects or interactions.

#### 3.1.4. Total visit duration

**Figure 3:**
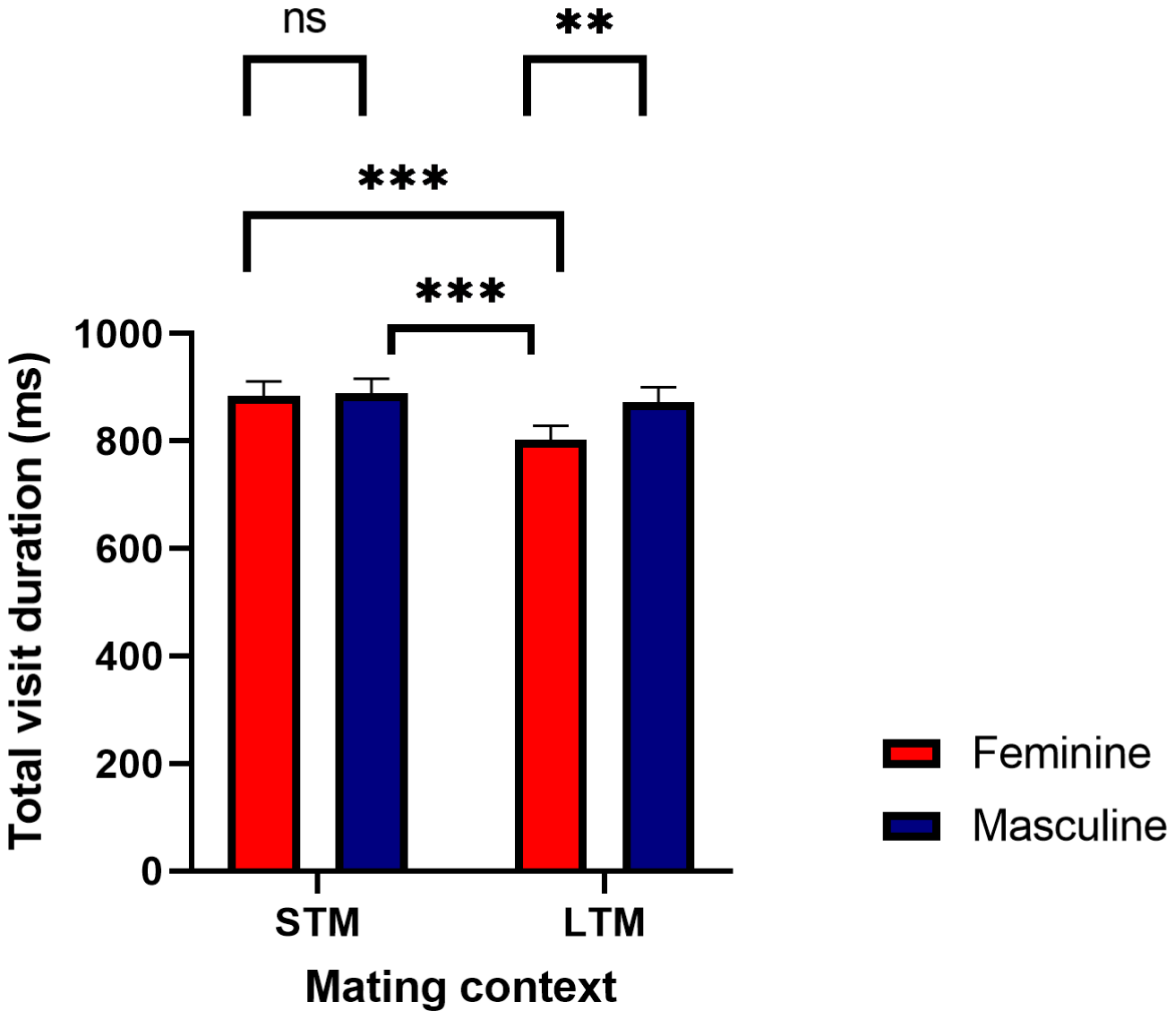
Women’s total visit duration in milliseconds as a function of facial masculinity and mating context. *p < 0.05, **p < 0.01, ***p < 0.001.

**Figure 4:**
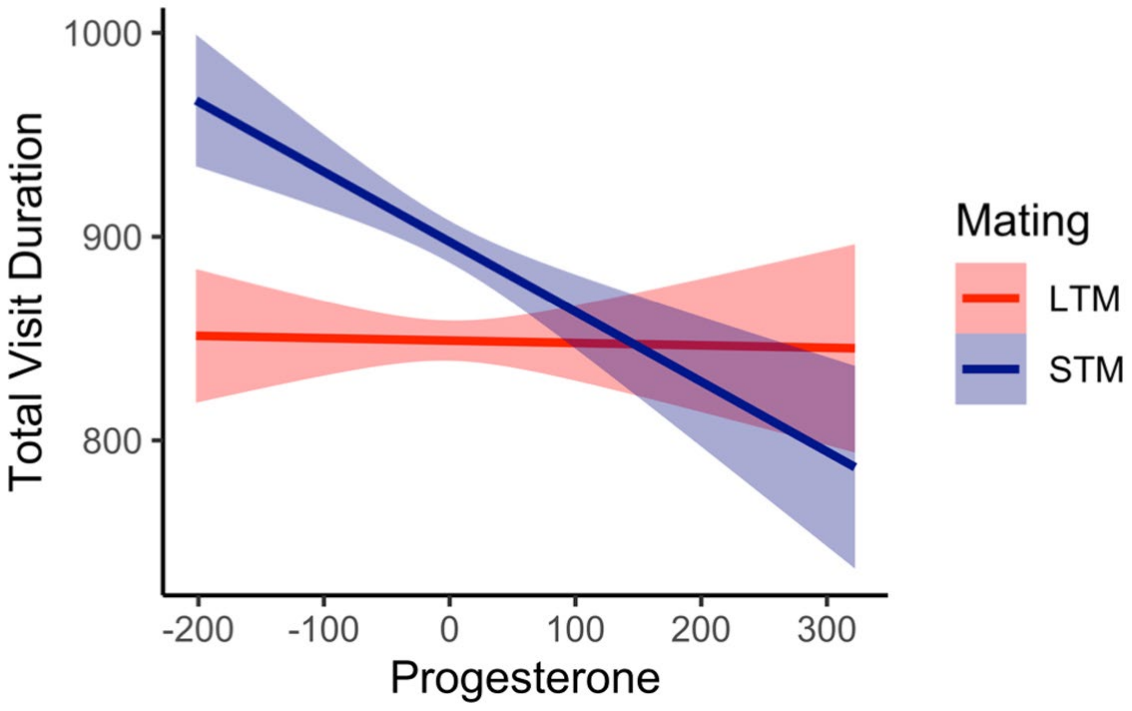
Interaction between women’s progesterone levels (cluster mean centered) and mating context predicting total visit duration in milliseconds.

Total visit duration was defined as the average amount of time spent viewing each region of interest (i.e., feminine, masculine face). The overall variance explained from the model was, *R*^2^_Marginal_ = 0.02, and R^2^_Conditional_ = 0.24. There was a significant effect for facial masculinity, *b* = 71.44, *SE* = 19.80, 95%CI [32.64, 110.28], *p* < 0.001. Pairwise comparisons revealed that women viewed masculine faces longer (*M* = 881, *SE* = 25) compared to feminine faces (*M* = 843, *SE* = 25). A main effect for mating context was significant, *b* = 85.73, *SE* = 19.91, 95%CI [43.16, 121.22], *p* < 0.001. Women viewed men longer when considering them for a short-term mating context (*M* = 887, *SE* = 25) compared to a long-term mating context (*M* = 838, *SE* = 24.9). This was further qualified by a facial masculinity by mating context interaction, *b* = −65.98, *SE* = 28.10, 95%CI [−112.11, −10.96], *p* = 0.02. There were significant differences when viewing men’s faces as a function of mating context, where women viewed masculine faces longer in a long-term mating context (*M* = 873, *SE* = 26.8) compared to feminine faces (*M* = 802, *SE* = 26.8). When considering a short-term mating context, women viewed feminine faces (*M* = 884, *SE* = 27.2) and masculine faces (*M* = 889, *SE* = 27.2) longer compared to viewing feminine faces in a long-term mating context (*M* = 802, *SE* = 27.2), see Figure 3. There was a significant interaction between mating context and women’s progesterone levels, *b* = −0.39, *SE* = 0.08, 95%CI [−0.47, −0.14], *p* < 0.001. The interaction was probed at −1SD, the mean, and + 1SD from the mean of progesterone. At lower levels (*b* = 79.42, *SE* = 16.33, 95%CI [47.14, 111.71], *p* < 0.001) and at the mean of progesterone (*b* = 49.17, *SE* = 14.08, 95%CI [21.11, 77.33], *p* < 0.001), women’s visual attention was higher for a short-term mating context compared to a long-term mating context. At higher levels of progesterone (*b* = 18.92, *SE* = 16.33, 95%CI [−13.35, 51.20], *p* = 0.24), the slopes were not significantly different from each other, see Figure 4. For long-term mating, women’s visit duration remained stable in relation to progesterone levels. Overall, women viewed masculine faces longer during a long-term mating context, and visual attention was partly associated with progesterone.

#### 3.1.5. Fixation count

Fixation count was defined as the number of times a visit (i.e., eye movement) was made into a region of interest (i.e., feminine, masculine faces). The overall variance explained from the model was, *R*^2^_Marginal_ = 0.007, and *R^2^*_Conditional_ = 0.26. There was a significant effect for facial masculinity, *b* = 0.25, *SE* = 0.10, 95%CI [0.02, 0.42], *p* = 0.03. Women made more visual fixations to masculine faces (*M* = 3.09, *SE* = 0.10) compared to feminine faces (*M* = 2.93, *SE* = 0.09). There was a significant effect for estradiol, *b* = 0.08, *SE* = 0.02, 95%CI [0.02, 0.11], *p* = 0.003. When women’s estradiol was higher they made more visual fixations.

## 4. Discussion

The current study investigated women’s visual attention to facial masculinity across their menstrual cycle. Our first hypothesis, that women with high E/P ratios would rate masculine faces more attractive and view them longer compared to feminine faces, was not supported. Masculine faces were not rated more attractive than feminine faces, and there were no interactions with E/P ratio, or estradiol and progesterone individually. Our second hypothesis, that visual attention to masculine faces would be moderated by mating context, where women with high E/P ratios would rate masculine faces as more attractive and view them longer during a short-term mating context, was not supported. Although there were no significant effects for attractiveness, women did view masculine faces longer compared to feminine faces but for a long-term mating context. Overall, feminine faces for a long-term mating context were given the least amount of visual attention, even when compared to masculine and feminine faces in a short-term mating context. In addition, there was no support for women with high E/P ratios viewing masculine men longer under a short-term mating context. Instead, there was partial support for the association between women’s hormones (estradiol, progesterone, and E/P ratio) and visual time. When women’s E/P ratio was high, they made fewer first fixation durations compared to when their E/P ratio was low. Further, when women’s progesterone levels were low, their visual attention was higher when considering men’s faces for a short-term mating context compared to a long-term mating context. In addition, there were differences in the number of visual fixations when looking at women’s hormones. When women’s estradiol was high, they made more visual fixations throughout.

The findings of the current study do not support previous work suggesting that facial masculinity is associated with ratings of attractiveness (Cunningham et al., 1990; Grammer and Thornhill, 1994; DeBruine et al., 2006; Little et al., 2007). This does not imply that facial masculinity is not important in women’s mate preferences, as women have shown that their overall preferences to masculinity may be different than what they choose (Flegr et al., 2019). In using a mating context prompt, we did not find any differences in attractiveness for women rating men under different mating contexts. Recent research has reported mixed results in facial masculinity preferences across mating contexts. Women have demonstrated preferences for all levels of facial masculinity when rating men for a co-parent compared to a short-term relationship (Stower et al., 2020). Others have shown a preference for facial masculinity for rating men for a long-term compared to a short-term mating context (Clarkson et al., 2020), consistent with the present findings.

In addition to ratings of attractiveness, the current study utilized sensitive gaze recording techniques (i.e., eye tracking) that may be able to capture subtle changes in attention to sexually dimorphic faces as a function of mating context and hormones. We did find that women’s late-onset visual measures (i.e., total visit duration, fixation count) were longer for masculine faces rather than feminine faces. Further, we found that women’s total visit duration was associated with facial masculinity and mating context, where women viewed masculine faces compared to feminine faces longer during a long-term mating context. This finding is surprising, given that we expected women to view masculine faces longer under a short-term mating context, as predicted by sexual strategies theory. This may indicate that when considering a partner under repeated occasions, women prioritize masculine features when considering a long-term relationship. Since short-term mating is meant to indicate a one-time sexual encounter, viewing men’s faces repeatedly in three different sessions eliminates the saliency of short-term mating, and long-term mating becomes more salient due to repeated exposure. Sexual strategies theory also suggests that women may prioritize men who are able to acquire resources and invest those resources in parenting, and facial masculinity has been perceived as being associated with the ability to protect (Sell et al., 2009). Further, this finding may be interpreted as women being attentive to features that connote both indirect (i.e., good genes) and direct benefits (i.e., protection, resource acquisition & transmission), which may be ideal attributes in seeking a partner for a long-term commitment. Little et al. (2007) demonstrated similar preferences when using a forced choice technique, where masculine preferences were higher for women considering men for a long-term partnership; however, in their study women were also taking into consideration ecological harshness, a condition not tested in this study. Given that women were simply viewing paired images of men’s faces, visual attention may not necessarily indicate that women found masculine faces more attractive, as demonstrated by the numerical ratings of attractiveness provided. Preferences for masculinity has been linked to women relying on heuristics to discern facial profiles, primarily in societies with highly developed health indices, such as the United States (Scott et al., 2014). It is plausible that women were relying on heuristics to discern facial masculinity, as testosterone levels may be higher in industrialized societies (Scott et al., 2014). Considering that facial masculinity has been associated with aggression, visual attention to masculine faces could possibly reflect attention to threatening faces or males that are successful in intrasexual competition (Puts, 2010; Scott et al., 2014; Boothroyd et al., 2015; Mefodeva et al., 2020). Overall, feminine faces for a long-term mating context were given the least amount of visual attention. Perhaps, in the context of long-term mating (i.e., commitment, marriage), facial femininity in men may not be a salient attribute and may not be given a considerable amount of attention compared to facial masculinity in a long-term mating context, and facial femininity and masculinity in a short-term mating context. It could also suggest that there is more attention given overall to faces when considering a short-term mating context, as women may be relying on underlying features associated with the best fit male and spend more time scanning men’s faces. Since women may prioritize high-quality genes in a short-term mating context (Buss and Schmitt, 1993), they may expend more cognitive resources in assessing potential mates for a context where immediate sexual access may occur.

The results of the study did not find any evidence for the ovulatory shift hypothesis, that women shift their preferences to men with putative markers of genetic quality (i.e., masculinity) across the menstrual cycle, supporting recent studies (Jones et al., 2018; reviewed in Marcinkowska et al., 2018a; Jones et al., 2019). The lack of evidence may have to do with the limited number of salivary samples (e.g., 3 in this study) provided and not confirming ovulation. Using an eye-tracking paradigm, which is meant to capture subtle movements in visual attention, we did not find evidence that women’s preferences to facial masculinity, either by attractiveness ratings or tracking eye movements, were associated with high E/P ratios.

The study includes limitations that future studies can investigate and build upon using an eye tracking paradigm. First, although we did collect three time periods across the menstrual cycle, in order to capture true conception probability, research should investigate fertility status by collecting daily salivary samples in addition to luteinizing hormone kits to confirm ovulation in women. In this study, women’s E/P ratio lacked heterogeneity, only showing slight changes across the three sessions. This impacted our ability to test if changes in E/P ratio was associated with shifting preferences and visual attention to facial masculinity, therefore, our results in reference to E/P ratio should be taken with caution. By testing women daily or including more than three samples, researchers may be able to capture heterogeneity in women’s reproductive hormones and be able to test accurately the ovulatory shift hypothesis using an eye-tracking paradigm. One possible explanation of the lack of heterogeneity in our sample is due to the study’s requirements and restrictions. We only recruited participants who had normal menstrual cycles and did not specify a particular range of cycle length (e.g., 23–35 day cycle length), nor did we assess average cycle length. Since “normal menstrual cycle” could have been broadly construed, future work should account for collecting women’s information of cycle length. Second, relying on a non-diverse sample limits what can be tested according to theoretical frameworks proposed in trade-offs made by women. That is, it is unclear if college students are making similar trade-offs in choosing a masculine partner for a short or a long-term relationship. Ecological constraints, such as diverse socioeconomic statuses and different life histories make more salient trade-offs compared to women attending a university. University women have a dense mating pool and are surrounded by cues of safety, where seeking a mate that may demonstrate physical features associated with protection (i.e., masculinity) may not be a priority according to their local ecology. Women have shown to calibrate their preference to facial masculinity dependent upon whether they are experimental (Little et al., 2007) or actual constraints (Marcinkowska et al., 2018b). Given these differences in life histories, it is unclear if overall preferences for facial masculinity are due to their indications of putative indicators of high-quality genes or successful intrasexual competition (Puts, 2010; Little et al., 2013). Masculine traits, such as beardedness, are preferred by women in populations where beards are frequent (Dixson et al., 2017) and where the sex ratio is male-biased (Dixson et al., 2019a). Bearded men are also considered as having higher parenting abilities (Dixson and Brooks, 2013), primarily among women with young children (Dixson et al., 2019b). Women have also shown preferences to bearded men because it may signal their ability to provide direct benefits in the form of immediate resources and protection. Further research may consider the overall perceptions of facial masculinity to indicate inter- or intrasexual selection traits. Lastly, we only included women who self-identified as exclusively heterosexual, and did not sample women with other sexual orientations (i.e., homosexual, bisexual). It is possible that women from this heterosexual sample could have a more nuanced sexual orientation, that could have been measured more precisely using a valid sexual orientation instrument. One study showed that women differ in their visual assessments of female bodies according to their sexual fluidity, showing similar patterns on how men view women (Widman et al., 2021). It is recommended future work incorporate a valid measure of sexual orientation in assessing attractiveness and visual assessments.

This study contributes to the overall literature on human mate choice by providing direct tests of sexual strategies theory and the ovulatory shift hypothesis. It introduces a different approach in studying the nuanced factors associated with women’s cyclic shifts and preferences for sexually dimorphic features. By using an eye tracking paradigm, the overall goal was to determine if the ovulatory shift hypothesis could be supported using real-time, implicit measures, by tracking eye movements to men’s facial masculinity. Although there was no strong evidence to suggest that women’s visual attention shifted when E/P ratios were higher, there were indications that women’s hormone levels (i.e., estradiol, progesterone, E/P ratio) predicted visual time to faces. This suggests these biomarkers play a supportive role in evaluating men’s faces under a particular mating context. However, it is important to interpret the findings using the E/P ratio with caution, as the study did not have enough variability in E/P ratio to make solid predictions about changes in that hormone measurement and its association with attractiveness and visual attention. Although, it is very unlikely that women will view the same male with feminine and masculine facial traits in an actual mating context, women do make quick decisions when considering potential mates in a real-world setting, such as in social gatherings, using mobile base dating apps (e.g., Tinder), and in speed dating (Todd et al., 2007).

In summary, women’s visual attention to facial masculinity was longer compared to feminine faces for a long-term mating context. We did not find any evidence to suggest that hormones were associated with visual attention to facial masculinity, but there was support that hormones are associated with overall visual attention. This indicates that there are biological underpinnings to the way that women strategically view men when considering them for a potential mate. Further work is needed to disentangle the role of hormones and their role in the cognitive processes of mate choice. Expanding work on the cognitive processes in mate choice can further the field by understanding the attentional processes in facial evaluations and by considering the biological underpinnings associated in mate preferences.

## Data availability statement

The raw data supporting the conclusions of this article will be made available by the authors, without undue reservation.

## Ethics statement

The studies involving human participants were reviewed and approved by the Oklahoma State University, Institutional Review Board. The patients/participants provided their written informed consent to participate in this study.

## Author contributions

RG and JB-C conceived and designed the study and wrote the first draft of the manuscript. RG collected the data and performed the statistical analysis. All authors contributed to manuscript.

## Conflict of interest

The authors declare that the research was conducted in the absence of any commercial or financial relationships that could be construed as a potential conflict of interest.

## Publisher’s note

All claims expressed in this article are solely those of the authors and do not necessarily represent those of their affiliated organizations, or those of the publisher, the editors and the reviewers. Any product that may be evaluated in this article, or claim that may be made by its manufacturer, is not guaranteed or endorsed by the publisher.
